# A Theory of Social Agency for Human-Robot Interaction

**DOI:** 10.3389/frobt.2021.687726

**Published:** 2021-08-13

**Authors:** Ryan Blake Jackson, Tom Williams

**Affiliations:** MIRRORLab, Department of Computer Science, Colorado School of Mines, Golden, CO, United States

**Keywords:** politeness theory, moral agency, human-robot interaction, social agency, levels of abstraction

## Abstract

Motivated by inconsistent, underspecified, or otherwise problematic theories and usages of *social agency* in the HRI literature, and leveraging philosophical work on *moral* agency, we present a theory of social agency wherein a social agent (a thing with social agency) is any *agent* capable of *social action* at some *level of abstraction*. Like previous theorists, we conceptualize *agency* as determined by the criteria of interactivity, autonomy, and adaptability. We use the concept of *face* from politeness theory to define *social action* as any action that threatens or affirms the face of a *social patient*. With these definitions in mind, we specify and examine the levels of abstraction most relevant to HRI research, compare notions of social agency and the surrounding concepts at each, and suggest new conventions for discussing social agency in our field.

## 1 Introduction and Motivation

The terms “social agency” and “social agent” appear commonly within the human-robot interaction (HRI) research community. From 2011 to 2020, these terms appeared in at least 45 papers at ACM/IEEE International Conference on HRI alone,^1^ with more instances in related conferences and journals. Given the frequency with which these terms are used in the HRI community, one might expect the field to have established agreed upon definitions to ensure precise communication. However, when these terms are used, they are often not explicitly defined, and their use frequently varies in important but subtle ways, as we will discuss below. Most HRI research is not concerned with exploring the entire philosophy of agency to find a theory that fits their study. As we show in Section 1.3, it is therefore common to simply use terms like “social agency” without espousing a particular concrete definition and move on under the assumption that it is clear enough to the reader what is meant. This may be fine within any individual paper, but confusion arises when different papers in the same research area use the same term with different meanings. We seek to formalize social agency in accordance with the existing underspecified usage because 1) having a rigorously specified definition for the term will help create common ground between researchers, help new researchers understand the vernacular of the community, and provide writing guidelines for HRI publications concerning social agency; and 2) attempting to redefine social agency in a substantially different way from existing habits of use would greatly hamper popular acceptance of the new definition.

**FIGURE 1:**
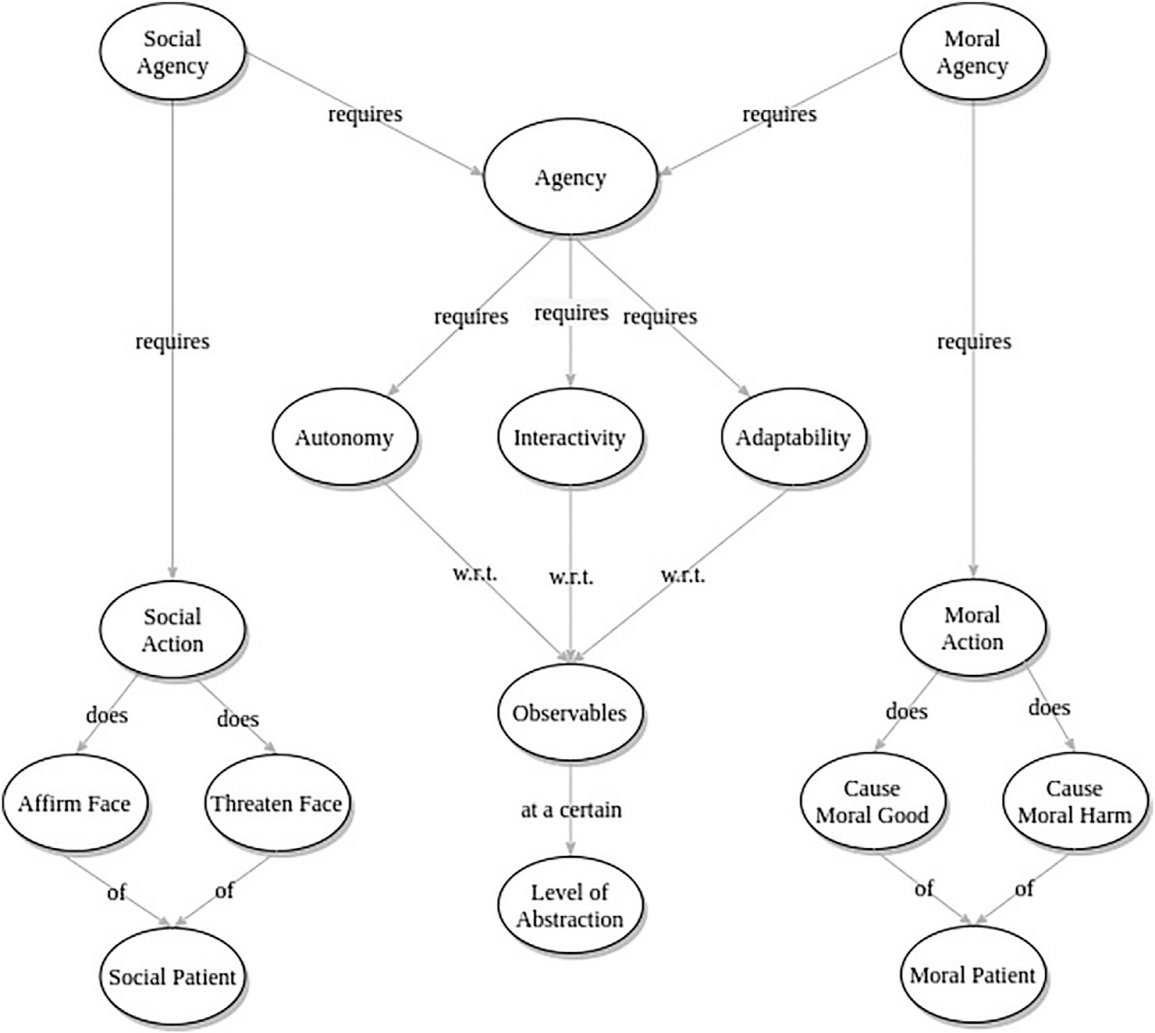
Concept diagram visualizing the theory of Social Agency presented in this paper, and the core concepts combined to construct this theory.

We present a theory of social agency for HRI research (as visualized in Figure 1) that deliberately aligns with and builds on other philosophical theories of robot agency. Specifically, we leverage insights from philosophers seeking to define *moral* agency in HRI. Moral agency provides an excellent analog to facilitate our discussion of social agency because it is an intimately related concept for which scholars have already developed rigorous definitions applicable to HRI, in a way that has not yet been done for social agency.

To design and justify our theory of social agency, we will first briefly survey existing definitions of social agency outside of HRI, and explain why those definitions are not well-suited for HRI. We will then survey theories of social agency from within HRI, and explain why those definitions are both inconsistent with one another and insufficient to cover the existing casual yet shared notion of social agency within our field. To illustrate this existing notion, we will then present a representative sample of HRI research that refers to social agency (without focusing on developing a definition thereof) to demonstrate how the greater HRI community’s casual use of social agency differs from the more rigorous definitions and theories found within and beyond the field of HRI.

### 1.1 Social Agency Outside Human-Robot Interaction

There are many different definitions of social agency from various disciplines including Psychology, Education, Philosophy, Anthropology, and Sociology. Providing an exhaustive list of these differing definitions is infeasible, but this section briefly summarizes a few representative definitions from different fields to show that they are not well-suited to HRI and to illustrate the broader academic context for our discussion of social agency.

Educational psychologists have used the term “social agency theory” to describe the idea that computerized multimedia learning environments “can be designed to encourage learners to operate under the assumption that their relationship with the computer is a social one, in which the conventions of human-to-human communication apply” (Atkinson et al., 2005). Essentially, social agency theory posits that the use of verbal and visual cues, like a more humanlike than overtly artificial voice, in computer-generated messages can encourage learners to consider their interaction with the computer to be similar to what they would expect from a human-human conversation. Causing learner attributions of social agency is hypothesized to bring desirable effects, including that learners will try harder to understand the presented material (Atkinson et al., 2005). In contrast, typically in HRI to be a social agent is humanlike in that humans are social agents, but more human-likeness, particularly in morphology or voice, does not necessarily imply more social agency. This theory also seems fundamentally concerned with social agency creating a social partnership to facilitate learning, but we also view non-cooperative social behaviors, like competition or argument, as socially agentic (Castelfranchi, 1998).

Other education researchers use the term social agency differently. For example, though Billett (2008) does not explicitly define social agency (a practice that we will see is common in HRI literature as well), they seem to view social agency as the capacity for the greater social world to influence individuals. This concept contrasts with personal agency, which Billett defines explicitly as an individual’s intentional actions. Personal and social agencies exert interdependent forces on the human worker as they negotiate their professional development and lives. This notion of social agency that precludes it from being a property held by a single individual, which does not seem to be how we use the term in HRI.

Scholars in education and social justice have also defined social agency as the extent to which individuals believe that being active socio-politically to improve society is important to their lives, and the extent to which individuals believe that they can/ought to alter power relations and structural barriers (Garibay, 2015; Garibay, 2018). This definition is largely centered around value placed on prosocial behavior. In contrast, in HRI we often apply the concept of social agency regardless of whether a robot is having any nontrivial impact on society or is trying to do so. We also ascribe social agency regardless of what a robot believes or values, or whether it can even believe or value anything.

Much of the discussion around agency in Anglo-American philosophy has revolved around intentionality, but some influential anthropologists have centered not only intentionality in defining agency, but also the power, motivation, and requisite knowledge to take consequential action (Gardner, 2016). Social agency, then, could be understood as agency situated within a social environment, wherein agents produce and reproduce the structures of social life, while also being influenced by those structures (and other material conditions), particularly through the rules, norms, and resources that they furnish. Social agency here is concerned with structures and relationships of power between actors. Other scholars in anthropology and related fields have criticized this notion of agency, for, among other reasons, over-emphasizing the power of the individual and containing values particular to men in the modern West. Some scholars that have de-emphasized power and capacity have stated that intentions alone are what characterize an agent and choices are the outcomes of these intentions, without necessarily qualitatively redefining the relationship between agency and social agency (Gardner, 2016). These definitions, and other similar ones, are also common in sociology and other social sciences. For reasons that we will argue below, we avoid “internal” factors like intentionality, motivation, and knowledge in defining social agency for HRI. We are also not concerned with whether robots have the power to act with broad social consequences since that does not seem important to HRI researcher’s usage of the term.

Anthropologists and archaeologists apply “social agency theory” to the study of artifactual tools and technologies to understand the collective choices that were made during the manufacture and use of such artifacts, the intentions behind those choices, the sociocultural underpinnings of those intentions, and the effects that the technologies had on social structures and relations. In doing so, they commonly refer to the social agency of technology or of technological practice to discuss the relationships between a technology and the social structures and decisions of its manufacturers and users. For example, the choice to use inferior local materials for tools rather than sourcing better materials through commerce given the material means to do so can indicate constraining social structures outweighing the enabling economic structures (Dobres and Hoffman, 1994; Gardner, 2016). Contrastingly, in HRI robots are discussed as having social agency in and of themselves, separate from that of the humans that make and use them. Social robots are also attributed social agency without really being embedded in the same broader social structures as their human interactants, though it is likely that they will be increasingly as the field progresses.

Scholars in Sociology have also conceptualized agency as the constructed authority, responsibility, and legitimated capacity to act in accordance with abstract moral and natural principles. Modern actors (e.g., individuals, organizations, and national states) have several different sorts of agency. Agency for the self involves the tendency of an actor towards elaborating its own capacities in accordance with wider rationalized rules that define its agency, even though such efforts are often very far removed from its immediate raw interests. For example, organizations often develop improved information systems toward no immediate goal. Agency for other actors involves opining, collaborating, advising, or modeling in service of others. Agency for nonactor entities is the mobilization for culturally imagined interests of entities like ecosystems or species. Finally, agency for cultural authority describes how, in exercising any type of agency, the actor assumes responsibility to act in accordance with the imagined natural and moral law. At the extreme, actors can represent pure principle rather than any recognized entity or interest. However, for the modern actor, being an agent is held in dichotomy with being a principal, where the principal “has goals to pursue or interests to protect, [and] the agent is charged to manage this interestedness effectively, but in tune with general principles and truths.” In other words, the principal is concerned with immediate raw interests, while the agent is concerned with higher ideals. For example, the goals of a university as principal are to produce education and research at low cost, whereas the goals of the university as agent include having the maximum number of brilliant (expensive) professors and the maximum number of prestigious programs. The same tension manifests in individuals as classic psychological dualisms (e.g., short-term vs. long-term interests) By this duality, highly agentic features like opinions and attitudes can be decoupled from behaviors, actions, and decisions (Meyer and Jepperson, 2000).

Social agency, within this body of work, refers to the social standardization and scriptedness of agency, and to how agency dynamics permeate and shape social structure. In a society of social agents, each individual or organization acts in accordance with their socially prescribed and defined agency, which is akin to the ideals defining their social role. In general terms, “the actorhood of individuals, organizations, and national states [is] an elaborate system of social agency…” wherein actors routinely shift between agency for the self and otherhood for the generalized agency of the social system. Individuals share in the general social agency of the system, negotiating the bases for their own existence via the rules and definitions of the broader system. This general social agency can function as the capacity for collective agentic action (Meyer and Jepperson, 2000). This understanding of agency as an upholding of higher ideals, principles, and truths (and social agency as the collective version of this), often in conflict with baser self-interested principalhood, is so different from conceptions of agency and social agency in HRI as to be essentially completely disjoint concepts. As we will illustrate below, agency in HRI is not (to our knowledge) discussed in duality with the notion of a principal, and social agency is not understood as a collective version of individual agency.

In presenting the definitions in this section, we do not intend to suggest that other fields have reached some sort of internal consensus regarding social agency or perfect consistency in its usage. Like in HRI, there appears to be ongoing conversation and sometimes disagreement about social agency within many fields, though the HRI-specific branch of this conversation seems relatively nascent. For example, there are ongoing debates in anthropology about whether (social) agency is an essential property of individuals, or somehow exists only in the relationships between individuals. Likewise, there are differing opinions within and between social science research communities about whether nonhuman entities can have (social) agency Gardner (2016). Unfortunately, we cannot present all perspectives here, nor can we really present the full detail and nuance of some of the perspectives that we *have* presented. What we hope to have indicated is that definitions of social agency from other fields, though academically rigorous and undoubtedly useful within their respective domains, are, for various reasons, neither intended nor suitable for the unique role of social agency in HRI, and an HRI-specific definition is needed.

### 1.2 Theories of Social Agency in Human-Robot Interaction

A number of theories of Social Agency have been defined within the HRI community to address the unique perspective of our field. Many of these grew out of foundational work on Social Actors from Nass et al. (1994), which suggested that humans naturally perceive computers with certain characteristics (e.g., linguistic output) as social *actors*, despite knowing that computers do not possess feelings, “selves”, or human motivations (Nass et al., 1994). This perception leads people to behave socially towards machines by, for example, applying social rules like politeness norms to them (Nass et al., 1994; Jackson et al., 2019). It is perhaps unsurprising that this human propensity to interact with and perceive computers in fundamentally social ways extends strongly to robots, which are often deliberately designed to be prosocial and anthropomorphised. While Nass et al.’s work establishing the theory that humans naturally view computers as social *actors* did not call computers “social agents” or refer to the “social agency” of computers, it nevertheless established that the human-computer relationship is fundamentally social, and laid the groundwork for much of the discussion of sociality and social agency in HRI today. In this section we will discuss four rigorously defined theories of Social Agency in HRI.

#### Nagao and Takeuchi

At around the same time that Nass and colleagues introduced their “Computers As Social Actors” (CASA) paradigm (Nass et al., 1994), Nagao and Takeuchi (1994) made one of the earliest references to computers as *social agents*. In describing their approach to social interaction between humans and computers, Nagao and Takeuchi argue that a computer is a social agent if it is both social and autonomous. These authors define socialness as multimodal communicative behavior between multiple individuals. Nagao and Takeuchi initially define autonomy as “[having] or [making] one’s own laws,” but later clarify that “an autonomous system has the ability to control itself and make its own decisions.” We will see throughout this paper that sociality and autonomy remain central to our discussion of social agency today, but not necessarily as defined by these authors.

Nagao and Takeuchi also define a social agent as “any system that can do social interaction with humans,” where a “social interaction” 1) involves more than two participants, 2) follows social rules like turn taking, 3) is situated and multimodal, and 4) is active (which might be better understood as mixed initiative). Some of these requirements, including at least the involvement of more than two participants and mixed initiativity, seem unique to this theory. Nagao and Takeuchi also differentiate their “social interactions” from problem solving interactions, though we believe, and see in the HRI literature, that task-oriented interactions can be social and take place among social agents.

#### Pollini

Pollini (2009) presents a theory that is less concerned with modality of interaction or type of robot embodiment, focusing instead on the role of human interactants in constructing a robot’s social agency. For Pollini, robotic social agents are both physically and socially situated, with the ability to engage in complex, dynamic, and contingent exchanges. Social agency, then, arises as the outcome of interaction with (human) interlocutors, as “the ability to act and react in a goal-directed fashion, giving contingent feedback and predicting the behavior of others.” We see the goal-directedness in this definition as loosely analogous to the notion of autonomy that is centered in other theories. In contrast to those theories, however, Pollini considers social agency as a dynamic and emergent phenomenon constructed collectively within a socially interacting group of autonomous actors, rather than as an individual attribute separately and innately belonging to the entities that comprise a social group. This presents a useful framing for understanding the social agency of multi-agent organizations like groups and teams. However, this multi-agent perspective prevents this definition from aligning with common references in HRI to the “social agency” of an individual robot. Nonetheless, some degree of autonomous behavior, interaction, perception, and contingent reaction must clearly remain central to our discussion of social agency.

Pollini also opines that “social agency is rooted in fantasy and imagination.” It seems that humans’ *attribution* of social agency may be tied to the development of imagination during childhood, leading Pollini to argue that people can “create temporary social agents” of almost anything with which they have significant contact, including toys like dolls, tools like axes, and places like the home. This leads them to the question “what happens when such ‘entities-by-imagination’ also show autonomous behavior and contingent reactions, and when they exist as social agents with their own initiative?” However, we argue that axes, dolls, and places actually *cannot* be social agents, at least not in the way that the typical HRI researcher means when they call a robot (or human) a social agent, since robots can conditionally take interactional behavior, which we believe is necessary for social agency.

Finally, Pollini argues that agency-specific cues embedded in robots (e.g., contingent behavior) are insufficient by themselves for creating social agency, and that social agency, rather, is negotiated between machines and their human interactants via a process of interpretation, attribution, and signification. This process involves interpreting a machine’s behavior as meaningful and explicative, and then attributing social agency based on the signification of that behavior as meaningful, which may also involve attributing internal forces like intentions and motivations. This means that, through this process, things with simple behaviors like cars or moving shapes on a screen can end up being ascribed social agency. Again, however, we see a fundamental difference between these examples and social robots, which can actually deliberately manifest meaningful and explicative behaviors. We interpret this discussion as circling the distinction between “actual” and “perceived” social agency that we will discuss below.

#### Levin, Adams, Saylor, and Biswas

Though much of the HRI literature exploring the standalone concept of *agency* is beyond the scope of this work as it focuses on the agency of machines without centering notions of *sociality*, the theory of agency from Levin et al. (2013) is relevant here because it explores attributions of agency specifically during social human-robot interactions. Levin et al. argue that people’s first impulse is to strongly differentiate the agency of humans and nonhumans, and that people only begin to equate the two with additional consideration (e.g., when prompted to do so by the robot defying initial expectations). They also describe how simple robot behavioral cues like the naturalness of movement or gaze can influence people’s attribution of agency to robots, as well as states and traits of the human attributor, like loneliness. Like some previous theories, Levin et al. center goal-orientedness and intentionality in their account of agency. However, they include not only behavioral intentionality, which we saw in other theories (Pollini, 2009), but also intentionality in cognition. Their example of this cognitive intentionality is drawing ontological distinctions between types of objects based on their use rather than their perceptual features.

#### Alač

Finally, Alač (2016) presents a theory in which multimodal interaction, situatedness, and materiality are important to a robot’s social agency, and justifies this theory with an observational study of a robot in a classroom. Alač frames robot agenthood as coexisting with the contrasting status of “thing,” with agentic features entangled in an interplay with a robot’s thing-like materiality. However, Alač moves away from discussing a robot’s social nature as an intrinsic and categorical property that resides exclusively in the robot’s physical body or programming, instead seeing robot sociality as enacted and emergent from how a robot is experienced and articulated in interactions. To Alač, the socially agentic facets of a robot are evident in the way it is treated by humans, focusing on proxemic and haptic interaction patterns and linguistic framing (e.g., gendering the robot) in group settings. Our work can augment ethnography-based theories like this one by exploring 1) the features of the *robot’s* behavior that give rise to perceptions of social agency, 2) what concepts constitute such perceptions, and 3) exactly what such perceptions imply. In other words, we focus on what social agency *is*, rather than on human behaviors that indicate ascription thereof.

### 1.3 Notions of Social Agency in Human-Robot Interaction

While in the previous section we discussed rigorously defined theories of social agency, much of the HRI literature that engages with social agency does not actually connect with those theories. In this section, we will thus explore the ways in which HRI researchers casually refer to social agency without focusing on developing or defining a formal theoretical account of it. Our goals in doing so are to 1) illustrate that notions of social agents and agency are commonly applied within the HRI research community, 2) provide examples of *how* these terms are used, and demonstrate important qualitative differences among the entities to which these terms are applied, 3) show that the existing theories defined in the previous section do not capture the common parlance usage of “social agency” among HRI researchers, and 4) lay the groundwork for developing a theory that does accommodate these usages.

There are many papers that refer to robots as social agents without mentioning or dealing with *social agency* per se. The term social agent is widely applied to entities that are both embodied (Heerink et al., 2010; Lee et al., 2012; Luria et al., 2016; Westlund et al., 2016) and disembodied (Lee et al., 2006; Heerink et al., 2010); remote controlled by humans (Heerink et al., 2010; Lee et al., 2012; Westlund et al., 2016) and self-controlled (Heerink et al., 2010); task-oriented (Heerink et al., 2010; Lee et al., 2012) and purely social (Lee et al., 2006); anthropomorphic (Heerink et al., 2010; Lee et al., 2012), zoomorphic (Lee et al., 2006; Heerink et al., 2010; Westlund et al., 2016), and mechanomorphic (Heerink et al., 2010; Luria et al., 2016); mobile (Heerink et al., 2010; Lee et al., 2012) and immobile (Heerink et al., 2010; Luria et al., 2016); and able to communicate with language (Heerink et al., 2010; Lee et al., 2012) and unable to do so (Lee et al., 2006; Luria et al., 2016). Any theory of social agency for HRI, then, should either encompass this diversity of social agents or account for ostensible misattributions of social agency. However, the theories we have examined, which emphasize embodiment (Nagao and Takeuchi, 1994; Alač, 2016), language (Nagao and Takeuchi, 1994), and self-control or intentionality (Pollini, 2009; Levin et al., 2013), exclude usages that are apparently common in HRI research.

Of course, one could argue that casual references to robots as “social agents” are synonymous to references to robots as “social actors,” and that such references do not actually have anything to do with the agentic nature of the robot. By this argument, the existing theoretical work on social agency in HRI would best be understood as investigating a completely separate topic from social agents. This reasoning, however, would result in a confusing state-of-affairs in which social agency is not a prerequisite for being a social agent, with the two topics unrelated except by the general connection to social interaction. We therefore assume that a social agent must be a thing with social agency, and that these two terms must be tightly and logically related. A clear conception of social agency is thus a prerequisite for the study of social agents. However, much of the work in HRI that concerns social agency does not focus on rigorously defining it. Indeed, some of these studies do not explicitly provide their definition of social agency at all.

An illustrative example of a casually referenced “social agent” is the “Snackbot” developed by Lee et al. (2012). The anthropomorphic Snackbot had real interactions with many humans over the course of multiple months as a snack delivery robot. The robot’s movement was self-controlled, but a human teleoperator hand-selected its delivery destinations. The human operator also remotely controlled the robot’s head and mouth movements and the robot’s speech, by selecting from a number of pre-made scripts, both purely social and task-oriented. We will refer back to this example in Section 2.

In their investigation of how cheating affects perceptions of social agency, Ullman et al. (2014) used perceptions of trustworthiness, intelligence, and intentionality as indicators of perceptions of social agency in an anthropomorphic robot. Using intentionality as a proxy for social agency aligns directly with several of the theories that we described in Section 1.2 (Pollini, 2009; Levin et al., 2013). Intelligence and trustworthiness, however, seem less closely related to social agency, and trustworthiness is explicitly not an aspect of social agency in theories that discuss competition and uncooperative behavior as inherently social actions (Castelfranchi, 1998).

Baxter et al. (2014) also study attributions of social agency to robots without explicitly defining the term, and measure it via a different proxy: human gaze behavior. This proxy does not obviously align with any of the theories of social agency discussed above. Although it is possible that gaze could be a good proxy for some definition of social agency (or the ascription thereof), further empirical work would be needed to establish that relationship.

Straub (2016) adopt yet another definition of social agency in their investigation of the effects of social presence and interaction on social agency ascription. In their study, social agents are characterized as “having an ‘excentric positionality,’ equipped with a) an ability to distinguish themselves, their perceptions as well as their actions from environmental conditions (embodied agency), b) the ability to determine their actions and perceptions as self-generated, c) having the ability to define and relate to other agents equipped with the same features of a) and b), along with d) defining their relationship to other agents through reciprocal expectations toward each other (‘excentric positioned’ alter ego).”

This definition, particularly part b, is somewhat ambiguous. One interpretation is that the robot simply needs to distinguish its own actions from the actions of others, and know that it is the cause for the effects of its actions; if the robot moves its arm into a cup, then it is the source for both the movement of the arm and the movement of the cup. However, this seems more like the robot knowing that its actions’ effects are self-generated and that it was the one that acted, rather than viewing the choice to act or the genesis of the action itself as self-generated. Another interpretation, which is similar to some of the definitions of social agency discussed in Section 1.1, is that seeing an action as self-generated requires the robot to understand its choice to act, perceive that choice as its own, and believe that it could have acted differently. This definition appears to require some form of consciousness or experience of free will, and is thus not well-suited to HRI. Straub uses human behavioral proxies, like eye contact, mimicry, smiles, and utterances, to measure ascriptions of social agency to robots (with more of these behaviors indicating more ascribed social agency), but such behavioral proxies do not measure all components of their definition.

Ghazali et al. (2019) study the effects of certain social cues (emotional intonation of voice, facial expression, and head movement) on ascriptions of social agency. Professedly inspired by research in educational psychology described above (Atkinson et al., 2005), they define social agency as “the degree to which a social agent is perceived as being capable of social behavior that resembles human-human interaction,” and then measure it by collecting participant assessments of the extent to which the robot was “real” and “like a living creature.” Roubroeks et al. (2011) use the exact same definition of social agency as Ghazali et al. (2019) in their investigation of psychological reactance to robots’ advice or requests, but operationalize it differently. Although they did not attempt to measure social agency, they did seek to manipulate it by varying robot presentation, presenting a robot’s advice as either text alone, text next to a picture of the robot, or a video of the robot saying the advice.

This definition seems problematically circular in that it defines social agency by the degree to which a social agent does something, without defining what it means to be a social agent. We also argue that Ghazali et al.’s chosen measures do not clearly align with the formal definitions of social agency proposed above, nor with Ghazali et al.’s stated definition. Moreover, this conceptualization excludes a large number of robots that the HRI literature calls social agents, and focuses on factors that many theories de-emphasize (e.g., livingness and human likeness). This example in particular shows that disparate definitions of social agency currently exist in the HRI literature, leading to confusion when authors underspecify or neglect to specify a definition.

Other work from Ghazali et al. (2018) on the relationship between social cues and psychological reactance centers the concepts of “social agent” and “social agency” explicitly, using the terms over 100 times in reference to robots and computers. However, the authors do not expressly provide any definition for those terms, despite ostensibly manipulating social agency in an experiment. Implicitly, the authors appear to follow their definition described above, with more humanlike superficial behavior (e.g., head/eye movement and emotional voice intonation) being considered more socially agentic, while the semantic content and illocutionary force of all utterances was kept constant across social agency conditions. However, Ghazali et al. (2018) also seem to consider the capacity to threaten others’ autonomy as a critical feature of social agency, since they measure perceived threat to autonomy as a manipulation check on social agency (though the social agency manipulation did not significantly impact perceived threat to autonomy). This choice was not extensively justified. As discussed in Section 2.2, perceived threat to autonomy is strongly related to (negative) face threat, which we view as important to social agency. However, as we will discuss, the capacity to threaten face is far broader than the capacity to threaten autonomy as measured by Ghazali et al. (2018).

To summarize, we have discussed several conflicting theories and usages of social agency in HRI, which, to varying extents: a) exclude common uses of the term “social agency” by being too restrictive, b) include objects that nearly all researchers would agree are neither social nor agentic, c) focus on factors that do not seem relevant to social agency in most pertinent HRI work, or d) conflate other concepts (like livingness or human-likeness) with social agency as it seems commonly understood. In addition, we have shown examples of the diversity of uses of the term “social agency” in the HRI research literature. We now contribute our own theory of social agency, with the specific intention of accommodating the HRI research community’s existing notions of social agency.

## 2 A Theory of Social Agency for Human-Robot Interaction

In this section, we propose a formal theory of social agency for HRI to address the challenges and limitations discussed in the previous sections. Our key arguments are: 1) social agency may be best understood through parallels to moral agency; 2) considering various levels of abstraction (LoAs) is critical for theorizing about any kind of agency; 3) a social agent can be understood as something with agency that is capable of social action; 4) social action is grounded in face; and 5) social and moral agency are related yet independent.

To best understand social agency, we draw parallels to recent work on moral agency. Not only are the concepts centered in theories of social agency discussed in Section 1.2 (e.g., autonomy, contingent behavior, and intentionality) also centered in many theories of moral agency, but the moral agency of robots and other artificial actors has also received a more rigorous treatment than social agency in the HRI literature. The moral agency literature thus represents a valuable resource for constructing a parallel theory of social agency. Furthermore, the two concepts of moral and social agency are inexorably linked, representing the two halves of interactional agency. They provide congruent relationships to (and means of understanding) moral/social norms and are key to our most foundational understandings of interaction. Given these similarities and connections, parallel understandings of the two concepts are not only intuitive but necessary, and we see no reason to attempt to define moral and social agency completely separately. For our purposes, we will leverage the moral agency theory of Floridi and Sanders (2004), but note that, as with social agency, there is not yet consensus among scholars as to a single canonical definition of moral agency, prompting ongoing debate (Johnson and Miller, 2008).

### 2.1 Agency and Levels of Abstraction

Because of historical difficulties in defining necessary and sufficient conditions for agenthood that are absolute and context-independent, Floridi and Sanders (2004) take analysis of *levels of abstraction* (LoAs) (Floridi, 2008) as a precondition for analysis of agenthood. A LoA consists of a collection of observables, each with a well-defined set of possible values or outcomes. An entity may be described at a range of LoAs. For a social robot, the observables defining an average user’s LoA might only include the robot’s behavior and other external attributes, like robot morphology and voice. In contrast, the robot developer’s LoA would likely also include information internal to the robot, such as the mechanisms by which it perceives the world, represents knowledge, and selects actions. Critically, a LoA must be specified before certain properties of an entity, like agency, can be sensibly discussed, as a failure to specify a LoA invites inconsistencies and disagreements stemming not from differing conceptions of agency but from unspoken differences in LoA.

The “right” LoA for discussing and defining moral agency must accommodate the general consensus that humans are moral agents. Floridi and Sanders (2004) propose a LoA with observables for the following three criteria: interactivity (the agent and its environment can act upon each other), autonomy (the agent can change its state without direct response to interaction), and adaptability (the agent’s interactions can change its state transition rules; the agent can “learn” from interaction, though this could be as simple as a thermostat being set to a new temperature at a certain LoA). For the sake of simplicity, we will consider LoAs consisting only of observations that a typical human could make over a relatively short temporal window. These observables encompass some concepts that were important to the theories discussed in Section 1.2 (e.g., autonomy and contingent behavior), and exclude others (e.g., teleological variables like intentionality or goal-directedness), which we discuss more below. We also consider a criterion that was *not* included in many theories for social agency, namely adaptability.

At the user’s LoA, wherein the deterministic algorithms behind a robot’s behavior are unobservable, the robot is interactive, autonomous, and adaptable, and therefore is an agent. However, at the robot developer’s LoA [or what Floridi and Sanders (2004) call the “system LoA”], which includes an awareness of the algorithms determining the robot’s behavior, the robot loses the attribute of adaptability and is therefore not an agent. These two LoAs will be important throughout the rest of this paper.

We argue that the distinction between these two LoAs (the user’s and the developer’s) explains why some scholars have suggested conceptualizing and measuring “*perceived* moral agency” in machines as distinct from moral agency itself. This notion of perceived moral agency would ostensibly capture “human attribution of the status of a machine’s agency and/or morality (independent of whether it actually has agency or morality)” (Banks, 2019), and these authors could easily define “perceived social agency” the same way.

Much of the impetus for defining these new concepts seems to be a desire to avoid the varied and conflicting definitions for agency (and the social and moral variants thereof). Typically within HRI, researchers are primarily concerned with how their robots are *perceived* by human interactants (the user’s LoA), and how those interactants might ascribe social agency to those robots. In that sense, perceived social agency as a concept seems like a good way to allow researchers to focus on what they really care about without getting mired in discussions of their robot’s “actual” agency, though it can still leave exactly what is perceived as (socially) agentic underspecified.

However, as we saw in Section 1, authors seldom refer to perceived social agency (particularly since we just defined it as parallel to perceived moral agency, which also does not seem to have caught on), but rather use the unqualified term “social agency”. Thus, rather than attempting to enforce a change in terminology, we propose that “perceived moral/social agency” should be understood as moral/social agency at the robot user’s LoA, and “actual” moral/social agency is the corresponding notion at the developer’s LoA. To illustrate, consider the SnackBot (Lee et al., 2012) described in Section 1.3. This robot was largely remotely controlled by a human, but, at the snack orderer’s (user’s) LoA it is a social agent. At the developer’s LoA, the robot is not an agent, but the system in aggregate might be considered socially agentic since one of its constituent parts, the human, is a social agent in and of itself.

If SnackBot could manifest the same behavior without human input, it would still not be agentic at the developer’s LoA insofar as its behavior is the direct result of deterministic algorithms that only act on its state. However, it does intuitively *seem* more agentic, prompting us to consider another useful LoA: one where we are aware of the general distributed system that controls a robot (in terms of software cognitive architectural components, hardware components like cloud computing, and human teleoperators), but not aware of the inner workings of each constituent part of that system. At this LoA, which we call the “architecture LoA”, a robot that does its computation internally might be agentic, but a robot that is remote controlled by either a person or another machine could not be an agent in and of itself. Hundreds of different LoAs could be constructed with various degrees of detail regarding how a robot works, but this is largely not constructive if humans are unlikely to ever view the robot from those LoAs. However, we believe that the architecture LoA is realistic for many potential robot interactants, particularly those that might own their own personal robots, or participants in laboratory HRI studies after the experimental debriefing.

At first glance, it would be easy to draw some parallels between our three main LoAs (developer’s, architecture, and user’s) and Dennett’s three stances from which to view an entity’s behavior in terms of mental properties (physical, design, and intentional) (Dennett, 1978). The user’s LoA in particular bears loose resemblance to Dennett’s intentional stance because the user is aware only of the robot’s externally observable behaviors, and may rationalize them by projecting internal states onto the robot. Likewise, our architecture LoA is explicitly concerned with the parts comprising a robot’s distributed system and the broad purpose of each constituent part, like the design stance, though it is not necessarily concerned with the purpose of the robot itself as a whole. However, several key distinctions separate our three LoAs form Dennett’s three stances. Most obviously, the developer’s LoA is unlike Dennett’s physical stance in that it is concerned with the algorithms producing the robot’s behavior but not the specifics of their implementation nor the hardware executing them.

More broadly, the three LoAs we have presented generally represent three of the *sets of information* that real people are most likely to have regarding robots during HRI, but there is no reason for this set of LoAs to be considered exhaustive, and no reason why our analysis of social agency cannot also apply to any other LoA from which a person views a robot. In contrast, more rigidly tripartite approaches to epistemological levelism, like Dennett’s, though readily formalized in terms of LoAs, contain an implicit ontological commitment and corresponding presupposed epistemological commitment because they privilege explanations over observable information (Floridi, 2008). That is not to say that such approaches to multi-layered analysis are not interesting and illustrative to HRI. For example, many researchers have explored whether humans naturally adopt the intentional stance towards robots and other artificial entities like they do towards other humans (Thellman et al., 2017; Marchesi et al., 2019; Perez-Osorio and Wykowska, 2019; Schellen and Wykowska, 2019; Thellman and Ziemke, 2019). However, it seems intuitive that robot developers versus users might take the intentional stance towards robots to different extents and under different conditions, so we posit that a specification of LoA is helpful in considering Dennett’s stances and other attitudinal stances in HRI in much the same way that it is to our discussion of social agency, rather than Dennett’s stances being homeomorphic to the three LoAs most salient here.

Most current cognitive architectures are precluded from agency at the developer’s LoA because any learning is typically a matter of updating the robot’s state by the deterministic rules of its code, rather than an actual update to the rules for transitioning between states (Floridi and Sanders, 2004). This includes black-box systems, like deep neural networks, because their lack of interpretability comes from an inability to fully understand how the state results in behavior, not from actual adaptability. However, we accept that humans have adaptability, and see no theoretical reason why the same level of adaptability could not be implemented in future artificial agents. Of course, particularly within the theory of causal determinism, there exists an LoA wherein humans do not have agency if all human behavior is rooted in the physical and chemical reactions of molecules in the brain (a “physical” LoA *a la* Dennett). Regardless of the veracity of this deterministic point of view, it seems clear that no LoA precluding agency from existing in the universe as we know it is a useful LoA at which to discuss agency in HRI.

We adopt the above notion of LoA and criteria for agenthood from Floridi and Sanders (2004) for our theory of social agency for several reasons. First, different LoAs help us to account for different understandings of social agency in the HRI literature, as we saw in our discussion of “actual” versus “perceived” social agency. Second, we can explicitly avoid conflating moral/social *agency* with moral/social *responsibility* (i.e., worthiness of blame or praise), which is another discussion beyond the scope of this paper. Third, avoiding internal variables like intentionality, goal-directness, and free-will guarantees that our analysis is based only on what is observable and not on psychological speculation, since a typical robot user cannot observe these attributes in the internal code or cognitive processes of their robot; we thus prefer a phenomenological approach.

Having established an understanding of agency, we now need to define some notion of sociality congruent to Floridi and Sanders’s notion of morality. However, we first want to point out that our justification for avoiding unobservable factors in defining and assessing (moral/social) agency parallels a similar argument from proponents of ethical behaviorism in defining and assessing the moral status of robots. Ethical behaviorism is an application of methodological behaviorism (as opposed to ontological behaviorism) to the ethical domain, which holds that a sufficient reason for believing that we have duties and responsibilities toward other entities (or that they have rights against us) can be found in their observable relations and reactions to their environment and ourselves. In other words, robots have significant moral status if they are roughly performatively equivalent to other entities that have significant moral status, and whatever is going on unobservably “on the inside” does not matter. This is not to say that unobservable qualia do not exist, nor do we deny that such qualia may be the ultimate metaphysical ground for moral status. However, the ability to ascertain the existence of these unobservable properties ultimately depends on some inference from a set of observable representations, so a behaviorist’s point of view is necessary to respect our epistemic limits (Danaher, 2020). We agree with this reasoning. Our definition of social agency could be framed as a form of “social behaviorism” that specifies the behavioral patterns that epistemically ground social agency and, by considering LoAs, is sensitive to the behaviors that are actually observed, rather than the set of behaviors that are, in principle, observable.

Of course, avoiding attributes like intentionality or goal directedness in our definitions in favor of a behaviorist approach does not completely free us from needing to rely on some form of inference. At a minimum, making observations from sensory input requires the inference or faith that one’s sensory inputs correspond to some external reality. Likewise, our interactivity criterion for agency requires some causal inference or counterfactual reasoning. For example, concluding that a robot can be acted on by the environment requires the counterfactual inference that the robot’s “response” to a stimulus would not have occurred absent that stimulus. Unfortunately, requiring some inference is unavoidable. In light of this, one could argue that it is equally reasonable and necessary to infer intention and goal directedness from behavior. For example, pulling on a door handle might signal an intent to open the door with the goal of getting into the building, even though the same behavior could also signal mindless programming to tug on handles without representing goals or having intentions. We argue that the sensory and causal inferences required by our framework are lesser epistemological leaps and more necessary and common (and therefore more justifiable) than inferences about other agent’s mental states like intentionality and goals. We also emphasize that goals and intentions are apparently not important to social agency at the developer’s LoA, since we saw many robots referred to as social agents by their developers in Section 1.3 that did not internally represent goals or intentions, and their developers would have known that.

### 2.2 Social Action Grounded in Face

We now move on to developing a notion of sociality congruent to Floridi and Sanders’s notion of morality. For Floridi and Sanders (2004), any agent that can take moral action on another entity (e.g., do good or evil; cause harm or benefit) is a moral agent. Any entity that can be the recipient of moral action (e.g., be harmed or benefited) is a moral patient. Most agents (e.g., people) are both moral agents and moral patients, though research has indicated an inverse relationship between perceptions of moral agency and moral patiency (e.g., neurodivergent adults are perceived more as moral patients and less as moral agents than neurotypical adults) (Gray and Wegner, 2009).

Just as a *moral* agent is any agentic source of moral action, we can define a *social* agent as any agentic source of social action. We ground our definition of social action in the politeness theoretic concept of “face” (Brown and Levinson, 1987). Face, which consists of positive face and negative face, is the public self-concept (meaning self-concept existing in others) that all members of society want to preserve and enhance for themselves. Negative face is defined as an agent’s claim to freedom of action and freedom from imposition. Positive face consists of an agent’s self-image and wants, and the desire that these be approved of by others. A discourse act that damages or threatens either of these components of face for the addressee or the speaker is a face threatening act. Alongside the level of imposition in the act itself, the degree of face threat in a face threatening act depends on the disparity in power and the social distance between the interactants. Various linguistic politeness strategies exist to decrease face threat when threatening face is unavoidable or desirable. Conversely, a face affirming act is one that reinforces or bolsters face for the addressee or speaker (though our focus will be on the addressee). We define social action as any action that threatens or affirms the addressee’s face. So, affirming and threatening face are social analogs to doing moral good and harm respectively. In contexts where it is helpful, this definition also allows us to refer to robots with different capacities to affect face as having different degrees of social agency, rather than viewing social agency as a strictly binary attribute. We also propose that the term “social *actor*” can refer to interactive entities capable of social action, but lacking the other criteria for agency (autonomy and/or adaptability).

Some scholars have opined that it is common to view social agents as equivalent to “communicating agents” (Castelfranchi, 1998), and thus might simply say that any communicative action is a social action. Though the ability to nontrivially communicate implies the capacity to threaten face, we choose to base our definition of social action directly on face because it allows for a more intuitive parallel to moral agency without excluding any meaningful communicative actions. The vast majority of communicative actions that an agent can perform have the capacity to impact face. Just in terms of face threat, any kind of request, reminder, warning, advice, offer, commitment, compliment, or expression of negative emotion threatens the addressee’s negative face, and any criticism, rebuke, insult, disagreement, irreverence, boasting, non-cooperation, or raising of divisive topics threatens the addressee’s positive face (Brown and Levinson, 1987). A single speech act can carry several elements that affect face in different ways, and even the mere act of purposefully addressing someone is slightly affirming of their positive face by acknowledging them as worth addressing, and slightly threatening of their negative face by imposing on their time. Indeed, it is difficult to think of a meaningful communicative action that would have no impact on face.

Another reason to ground social action in face is because face is more concrete and computationalizable than some other options (e.g., induced perceptions of human likeness or influence on emotional state), while still being broad enough to encompass the whole set of actions that we would intuitively consider to be social. There exist various parameterizations or pseudo-quantifications of face threat/affirmation, including Brown and Levinson’s own formula which presents the weight of a face threatening act (*W*) as the sum: *W* = *D*(*S*, *H*) + *P*(*H*, *S*) + *R* where *D*(*S*, *H*) is the social distance between the speaker (*S*) and hearer (*H*), *P*(*H*, *S*) quantifies the power that *H* has over *S*, and *R* represents the culturally and situationally defined level of imposition that the face threatening act entails. For negative face threatening acts, *R* includes the expenditure of time and resources. For positive face threatening acts, *R* is harder to determine, but it is given by the discrepancy between *H*’s own desired self-image and that presented in the face threatening act. Individual roles, obligations, preferences, and other idiosyncrasies are subsumed into *R*. Of course, the constituent parts of this equation cannot be precisely quantified in any canonical way (nor can, for example, influence on behavioral or emotional status). We do not view this as a weakness because we would not expect to precisely quantify the magnitude of socialness in an action. Humans cannot precisely answer questions like “How social is it to hug your grandmother?” or “Which is more social, asking a stranger for the time or tipping your waitress?”. However, this equation nonetheless illustrates some of the concrete underpinnings of face and shows how face connects to concepts like relational power, interpersonal relationships, material dependence, cultural mores, etc.

Robots are valid sources of social action under this face-based definition. Typical task-oriented paradigms of HRI involve robots either accepting or rejecting human requests (which either affirms or threatens both positive and negative face), or making requests of humans (which threatens negative face). Even simply informing human teammates about the environment threatens negative face by implying that the humans ought to act based on the new information. Less task-oriented cases, like companionship robots for the elderly (Heerink et al., 2010), also require face affecting social actions, though these may tend to be more face affirming than in task-based interaction. Again taking the SnackBot Lee et al. (2012) as an example, bringing someone a requested snack is face affirming, and so are dialogue behaviors like complimenting snack choice or apologizing for delays. The SnackBot’s dialogue behavior of asking people to move out of the way is face threatening. Research examining how robots influence human face and how humans react to robotic face threatening actions is ongoing (Jackson et al., 2019; Jackson et al., 2020).

In comparison to our definition, Castelfranchi (1998) define an action as either social or nonsocial depending on its purposive effects and the mind of the actor. Their social actions must be *goal-oriented* and motivated by *beliefs* about predicted effects in relation to some goal. Their social actions are mainly based on some exercise of power, to attempt to influence the behavior of other agents by changing their minds. They specifically say that social action cannot be a behavioral notion based solely on external description. This definition is not well-suited to our purposes because these internal underpinnings are unknowable to a typical robot user, and thus preclude the user from viewing a robot as a social agent. We saw similar reasoning in our decision to exclude goal-orientedness as a prerequisite for agency. Even if a user chooses to adopt an intentional stance (see Dennett, 1978) toward a robot and infer goals motivating its behavior, this does not imply that the robot actually has an internal representation of a goal or of the intended effects of its actions; the person’s intentional stance would only allow them to take social action towards the robot, not vice versa. Given the popular perception of robots as social and the academic tendency to call them social agents, we do not want a definition of social action that cannot apply to robot action or that relies on factors that cannot be observed from a user’s LoA. Furthermore, Castelfranchi’s definition excludes, for example, end-to-end deep neural dialogue systems that may not explicitly represent goals, beliefs, causality, or interactants as potential sources of social action, but whose actions can clearly come across as social and carry all the corresponding externalities. Our face-based definition does not have these limitations.

To be clear, our decision to define social action via face is not an arbitrary design choice, but rather a result of face’s integral role in all social interaction. We believe that an action’s relationship to face is, unavoidably and fundamentally, what determines whether that action is social because face is what creates the experience of having social needs/desires in humans. It follows that, for robots, the appearance or attribution of face, or some relationship to others’ face, is what allows them to be social actors. Any action that affects face is necessarily social, and any action that does not is necessarily asocial. This aligns well with widespread intuitions about sociality and common parlance use of the term.

### 2.3 Social Patiency as Having Face

Any social action must have a recipient whose face is affected. If social agency is an agent’s capacity to be a source of social action (to affirm or threaten face), then the corresponding notion of social *patiency* is the capacity to have one’s face threatened or affirmed (i.e., having face). This is similar to the notion of moral patiency as the capacity to be benefited or harmed by moral action. Clearly, conscious humans are simultaneously moral and social agents and patients at any reasonable LoA. However, neither moral nor social patiency at any given LoA strictly requires moral or social agency at the same LoA, which leads us to the question of whether our robotic moral/social agents in HRI are also moral/social patients.

It seems clear that, at a reasonable LoA for a human interactant, it is possible to harm a robot, making the robot a moral patient. This is especially clear for robots capable of affective displays of protest and distress (Briggs and Scheutz, 2014). Indeed people deliberately abuse robots with surprising frequency (Nomura et al., 2015). However, at a deeper LoA, we know that current robots cannot feel pain (or pleasure), have no true internal emotional response to harm like fear, and lack the will towards self preservation inherent in most lifeforms. Thus, at this deeper LoA the robot is not a moral patient.

Likewise, a robot’s social patiency depends on the LoA considered. It is feasible to program a robot to manifest behaviors indicating face wants, like responding negatively to insults and positively to praise, in which case it would be a social patient at the user’s LoA. However, at the developer’s LoA, the robot still has no face.

### 2.4 Social and Moral Agencies as Independent

We now discuss the extent to which social agency and moral agency can manifest in machines independent of one another. We believe that some machines, including some robots, are largely perceived as asocial moral agents, while others are seen as amoral social agents. Although, for the most part, social robots do not fall in either of these groups, we believe that they are worth presenting as points of reference for understanding the special moral and social niche occupied by language capable robots. We continue to consider these technologies from the user’s LoA.

Some artificial agents are popularly ascribed some form of moral agency without behaving socially or even possessing the capacity for communication outside of a narrow task-based scope. We call such agents “asocial moral agents”, and use autonomous motor vehicles as the quintessential example. If we include the likely possibility that autonomous vehicles will learn and change their behavior in response to changing road conditions or passenger preferences, they are agentic at the passenger’s LoA by being interactive, autonomous, and adaptive.

In terms of moral action, while autonomous motor vehicles are obligated to conform to the legal rules of the road, they are also expected to engage in extralegal moral decision making and moral reasoning. Myriad articles, both in popular culture and in academia, contemplate whether and how autonomous cars should make decisions based on moral principles (e.g., Bonnefon et al., 2016). Questions like “in an accident, should the car hit a school bus to save its own passenger’s life? Or should it hit the barrier and kill its passenger to save the school children?” have taken hold of popular imagination and proliferated wildly. Regardless of the actual usefulness of such questions (cf. Himmelreich, 2018), it is clear that autonomous cars are being ascribed moral agency.

We can also consider whether autonomous vehicles might be capable of social action. For example, using a turn signal is clearly communicative, but it is also legally mandated; an autonomous vehicle would signal an impending turn regardless of whether any other driver was present to see the turn signal. Given the legal motivation behind the turn signal and the fact that it has no specific intended addressee, we view it as the rare communicative act with no (or negligible) impact to face. Indeed, any communication via turn signal would be considered incidental to law-following by the typical driver. Other driving behavior can also be communicative; though we do not expect autonomous vehicles to engage in tailgating or road rage, we could imagine that they might change the norms governing human driving behavior by modeling those norms themselves. For example, if all autonomous vehicles on the road adopt a uniform following distance, this behavior might influence human drivers sharing the road to do the same. However, this potential normative influence is distinct from that of social robots in that it is passive, incidental, unintentional, and not principally communicative, and therefore not face-relevant.

In other cases, depending on behavior, robots could be perceived as amoral social agents. Social robots that do not have the ability to act on their environment in any meaningful extra-communicative capacity may be physically unable (or barely able) to produce moral action. As an example, consider MIT’s Kismet robot, which is expressive, (non-linguistically) communicative, and social, but largely helpless and incapable of acting extra-communicatively. Many social actions are available to Kismet. For example, making a happy expression/noise when a person enters the room is face affirming, and a disgusted expression face threatening. Given the right behaviors, Kismet could also meet our prerequisites for agency and be an amoral social agent.

When moral and social agency are both present, as is the case for most social robots at the user’s LoA, their combination gives rise to interesting phenomena. Social robots can occupy a unique sociotechnical niche: part technological tool, part agentic community member. This status allows robots to play an active role in shaping the community norms that inform human morality, which behavioral ethics has shown to be dynamic and malleable (Gino, 2015). And while robots are not the only technology to play a role in shaping human norms (Verbeek, 2011), we believe their social agency grants them uniquely powerful normative influence. For example, robots have been shown to hold measurable persuasive capacity over humans, both via explicit and implicit persuasion (Briggs and Scheutz, 2014; Kennedy et al., 2014), and even to weaken human (application of) moral norms via simple question asking behavior (Jackson and Williams, 2019).

Language capable robots are unique among technologies not only in the strength of their potential moral influence, but also in their ability to take an active and purposeful role in shaping human moral norms (or human application of moral norms) as social agents. However, this capability is a double-edged sword. On the one hand, robots of the future could productively influence the human moral ecosystem by reinforcing desirable norms and dissuading norm violations. On the other hand, today’s imperfect moral reasoning and natural language dialogue systems open the door for robots to inadvertently and detrimentally impact the human moral ecosystem through reasoning errors, miscommunications, and unintended implicatures. It is thus crucial to ensure moral communication and proper communication of moral reasoning from robots, especially in morally consequential contexts. The power to transfer or alter norms comes with the responsibility to do so in a morally sensitive manner.

## 3 Revisiting Related Work

Revisiting the theories of social agency from Section 1.2, we see that our definition is more inclusive than that of Nagao and Takeuchi (1994) and Alač (2016) in that we demphasize the robot’s embodiment and materiality to account for purely digital potential social agents that we see in HRI research (Lee et al., 2006; Heerink et al., 2010), and do away with the teleological and internal considerations (e.g., goal-orientedness and intentionality) that would not be knowable to the typical robot user (cp. Pollini, 2009; Levin et al., 2013). On the other hand, our work is more restrictive than Pollini (2009) because we exclude “entities by imagination” as potential social agents, and specify that there are several behavioral traits necessary for social agency. This approach balances the more human-ascription-centered and more robot-trait-centered conceptualizations of social agency. Our theory acknowledges the human role in determining social agency by centering human face and the human’s LoA, without reducing social agency to the mere ascription thereof. At the same time, we concretely describe the robot traits necessary for social agency at a given LoA.

Revisiting the studies from Section 1.3, which referenced social agents and social agency without principally focusing on defining those concepts, we see that our definition can encompass the wide diversity of potential social agents in HRI. Particularly at the user’s LoA, robots can be social agents regardless of embodiment, teleoperation, task-orientedness, morphology, mobility, or linguistic capacity. However, some of the robots we reviewed would actually be excluded by our definition at the user’s LoA by failing to meet behavioral prerequisites, particularly by lacking indications of adaptability (e.g., Lee et al., 2006; Heerink et al., 2010; Roubroeks et al., 2011). Interestingly, robots with a human teleoperator, like the SnackBot (Lee et al., 2012) might be *more* likely to be socially agentic at the user’s LoA than those with simpler self-controlled behavior.

Finally, we stress that our theory complements (rather than competes with) much of the previous work we discussed. For example, some of the proxemic and haptic human behavior that Alač (2016) observed in their ethnographic study, like the choice to touch a robot’s forearm rather than other body parts, might be understood within our theory as stemming from attributions of social *patiency* to the robot, rather than social agency. Likewise, our conception of social agency may well be tied to, for example, psychological reactance (Roubroeks et al., 2011) or trust (Ullman et al., 2014).

## 4 Concluding Remarks

We have presented a theory of social agency wherein a social agent (a thing with social agency) is any *agent* capable of *social action* at the *LoA* being considered. A LoA is a set of observables, and the LoAs most relevant to our discussion have been the robot user’s, the developer’s (or system LoA), and, to a lesser extent, the architecture LoA. *Agency* at any given LoA is determined by three criteria which we defined concretely above: interactivity, autonomy, and adaptability. We have defined *social action* as any action that threatens or affirms the addressee’s face, and refer to the addressee in this scenario as a social patient. More specifically, *social patiency* is the capacity to be the recipient of social action, i.e., having face. These definitions came from parallel concepts in the philosophy of *moral* agency (Floridi and Sanders, 2004). We motivated our theory of social agency by presenting a sample of the inconsistent, underspecified, and problematic theories and usages of social agency in the HRI literature.

Based on our theory, we have several recommendations for the HRI community. We recognize a tendency to casually use the word “agent” to refer to anything with any behavior, and to correspondingly use “social agent” to simply mean “social thing.” A summary of the concepts that are central to our theory can be found in Table 1. We encourage authors to consider either switching to the broader term “social actor” as defined above, or to briefly specify that they are using the term “social agent” informally and do not intend to imply social agency in any rigorous sense. We further recommend that any paper dealing with social agency be specific in selecting a suitable definition (such as the one presented in this work) and LoA.

**TABLE 1:** Summary of terms that are important to our concept of social agency.

Term	Definition
Level of Abstraction (LoA)	A collection of observables describing an entity (Floridi and Sanders, 2004; Floridi, 2008). A user’s LoA for a robot includes movement, speech, morphology, etc., while the developer’s LoA also includes the algorithms controlling the robot
Agent	Anything possessing the three criteria of interactivity, autonomy, and adaptability
Interactivity	The capacity to act on the environment and to be acted upon by the environment (Floridi and Sanders, 2004)
Autonomy	The capacity to change state without direct response to interaction (Floridi and Sanders, 2004)
Adaptability	The capacity for interaction to change the system’s state transition rules. The capacity to “learn” from interaction (Floridi and Sanders, 2004)
Social agent	Anything capable of taking social action at the LoA under consideration
Social action	Any act that threatens or affirms an other’s face. Analogous to moral action (doing harm/good to an other)
Social patient	Anything that can be a recipient of social action, i.e., anything with face
Face	The public self-concept (meaning self-concept existing in others) that all members of society want to preserve and enhance for themselves
	Negative face: an individual’s claim to freedom of action and freedom from imposition
	Positive face: an individual’s self-image and wants, and the desire that these be approved of by others (Brown and Levinson, 1987)

It will be important for future studies to develop, refine, and validate measurements of social (and moral) agency. There exists early work on developing a survey to measure “perceived moral agency” for HRI (Banks, 2019), however some questions seem to conflate moral *goodness* with moral *agency*, and, despite measuring facets of autonomy and moral *cognition*, the survey does not measure the capacity for taking moral *action*. Some of the proxies that we saw used for social agency in Section 1.3, like human-likeness, realness, and livingness (Ghazali et al., 2019) do not match our new conceptualization of social agency. Others, like gaze (Baxter et al., 2014), could be promising but have yet to be validated with our theory (or, to our knowledge, any particular theory) of social agency in mind. Validated metrics would facilitate experimental work motivated by our theory.

For example, future work designed to evaluate and further concretize our theory could empirically verify whether changing the LoA at which somebody is viewing a robot causes a corresponding change to their assessment of that robot as a (social) agent. The results could either strengthen the argument that the LoA is a critical prerequisite for the discussion of agency, or indicate that colloquial conceptions of agency do not account for LoA, despite its importance in rigorous academic discussions. Another avenue for this type of work would be to manipulate the magnitude of face threat/affirmation that a social robot is capable of and examine how that manipulation effects perceptions of the robot as a social agent. This experiment would specifically target our definition of social action as grounded in face.

Measures of social agency would also allow us to examine its relationship with persuasion and trust. On the one hand, we could imagine that decreasing a robot’s social agency (by lowering its propensity to affect face) could increase its persuasive capacity if people are more amenable to persuasion when their face is not threatened. On the other hand, increasing a robot’s social agency might increase its persuasive capacity if people are more likely to trust a more human-like robot.

Furthermore, it will be important to probe for causal relationships between ascriptions of social agency and ascriptions of moral responsibility and competence in robots. In human children, development of increased capacity for social action is typically correlated with development of other facets of intelligence and skills, including moral reasoning. However, this correlation does not necessarily exist for robots, since a robot could be socially agentic and competent, with a wide range of possible social actions, and still have no moral reasoning capacity. If robot social agency, or social behavior in general, leads interactants to assumptions of moral competence or overall intelligence (as it likely would in humans), this could lead to dangerous overtrust in robot teammates in morally consequential contexts that they are not equipped to handle. Thus, giving a robot linguistic/social competence would also necessitate giving the robot a corresponding degree of moral competence.

Finally, though there is evidence for an ontological distinction between humans and robots (Kahn et al., 2011), it is not yet clear where differences (and similarities) will manifest in terms of moral and social agency. We will require human points of reference in future HRI studies to fully understand how the emerging moral and social agency of robots relate to those qualities in humans.

## Data Availability

The original contributions presented in the study are included in the article/Supplementary Material, further inquiries can be directed to the corresponding author.

## Footnotes

1
https://dl.acm.org/action/doSearch?AllField=%22Social+agent%22+%22social+agency%22&ConceptID=119235
